# Broodstock History Strongly Influences Natural Spawning Success in Hatchery Steelhead (*Oncorhynchus mykiss*)

**DOI:** 10.1371/journal.pone.0164801

**Published:** 2016-10-13

**Authors:** Michael J. Ford, Andrew R. Murdoch, Michael S. Hughes, Todd R. Seamons, Eric S. LaHood

**Affiliations:** 1 Conservation Biology Division, Northwest Fisheries Science Center, National Marine Fisheries Services, National Oceanic and Atmospheric Administration, Seattle, Washington, United States of America; 2 Supplementation Research Team, Washington Department of Fish and Wildlife, Wenatchee, Washington, United States of America; 3 Molecular Genetics Laboratory, Washington Department of Fish and Wildlife, Olympia, Washington, United States of America; Ohio State University, UNITED STATES

## Abstract

We used genetic parentage analysis of 6200 potential parents and 5497 juvenile offspring to evaluate the relative reproductive success of hatchery and natural steelhead (*Onchorhynchus mykiss*) when spawning in the wild between 2008 and 2011 in the Wenatchee River, Washington. Hatchery fish originating from two prior generation hatchery parents had <20% of the reproductive success of natural origin spawners. In contrast, hatchery females originating from a cross between two natural origin parents of the prior generation had equivalent or better reproductive success than natural origin females. Males originating from such a cross had reproductive success of 26–93% that of natural males. The reproductive success of hatchery females and males from crosses consisting of one natural origin fish and one hatchery origin fish was 24–54% that of natural fish. The strong influence of hatchery broodstock origin on reproductive success confirms similar results from a previous study of a different population of the same species and suggests a genetic basis for the low reproductive success of hatchery steelhead, although environmental factors cannot be entirely ruled out. In addition to broodstock origin, fish size, return time, age, and spawning location were significant predictors of reproductive success. Our results indicate that incorporating natural fish into hatchery broodstock is clearly beneficial for improving subsequent natural spawning success, even in a population that has a decades-long history of hatchery releases, as is the case in the Wenatchee River.

## Introduction

Hatchery propagation is a ubiquitous element of Pacific salmon management, with hatchery-origin fish accounting for high proportions of adult spawning runs of several species returning to major systems such as the Sacramento and Columbia Rivers and rivers flowing into Puget Sound [1, 2]. Many long-term hatchery programs for Pacific salmon are designed to create separate artificially propagated populations as mitigation for natural populations lost due to lost access to spawning and rearing areas or other types of habitat degradation [3]. Starting in the late 1980’s, however, and increasing after widespread listings of Pacific salmon under the U.S. Endangered Species Act in the mid-1990s, hatchery programs in the Pacific Northwest and California have increasingly emphasized direct supplementation of at-risk natural populations with hatchery-produced fish in order to prevent extirpation and increase natural spawning abundance [3–5].

Despite decades of research, uncertainties remain about the effectiveness of supplementation as a conservation strategy [6]. Some studies have found increased natural population abundance attributed to supplementation (e.g. [7, 8]), but others have found small, no, or inconsistent effects (e.g. [9–11]). In addition, multiple studies have found reduced natural spawning success by hatchery origin salmon (reviewed by [12, 13]), and theoretical work suggests that loss of fitness due to inadvertent domestication selection is potentially a significant risk [14, 15].

Multiple studies have used genetic parentage analysis to estimate the reproductive success of individual natural and hatchery origin salmon spawning in the wild (reviewed by [12]), but to date only a few studies have explicitly measured the effects of broodstocking practices on the subsequent reproductive success of the naturally spawning progeny of fish spawned in hatcheries. In one influential study of steelhead (*Oncorhynchus mykiss*) in the Hood River, Oregon [16–20], researchers found that hatchery fish produced from broodstock consisting of one hatchery origin and one natural origin parent (H_HN_) had substantially lower reproductive success when spawning naturally than either natural origin fish or hatchery fish produced from broodstock consisting of two natural origin parents (H_NN_). This result is important because it indicates the existence of a potentially heritable reduction in fitness associated with hatchery propagation. An additional study on the same population [21] showed that this fitness reduction persists in the natural population for at least one generation, suggesting that the reduced fitness caused by hatchery rearing may cause fitness loss in the supplemented population.

To date, we are aware of only two other studies, one on Chinook salmon (*O*. *tshawytscha*) [22] and another on coho salmon (*O*. *kisutch*) [23], that have directly compared the reproductive success of H_NN_ and H_HN_ salmon when spawning in a natural stream. Although both studies found that hatchery origin fish had reduced natural spawning success compared to natural origin fish, neither study found a significant difference in reproductive success associated with broodstock origin (i.e., H_NN_ compared to H_HN_). Thus, considerable uncertainty remains about whether additional fitness reductions associated with use of second or more generation hatchery fish for broodstock are common, or are perhaps associated only with certain populations, species, or other situation-specific factors.

Here, we report on the results of a reproductive success study of hatchery and natural origin steelhead in the Wenatchee River, Washington. Like the earlier Hood River study, we used genetic parentage analysis to directly compare the reproductive success of natural origin fish and hatchery origin fish from broodstock consisting of zero, one or two natural origin parents (H_HH_, H_HN_, and H_NN_, respectively). We found that hatchery broodstock type had a significant influence on reproductive success in this population, replicating an important result of the earlier Hood River study.

## Methods

### Overview

Similar to other recent studies of salmon reproductive success, we employed a genetic parentage approach to estimate the number of progeny produced by individual spawners. Briefly, a high proportion of the adult spawning run was captured at a site downstream of the spawning areas of the population. Tissue was collected for genetic analysis, and the hatchery or natural origin of each potential spawner was determined, along with biological information such as sex, length, and weight. The fish were then released to continue their migration and spawn naturally. Subsequently, juvenile progeny of this spawning population were sampled throughout the watershed, and genetic information collected from the potential parents and progeny were used to estimate the relative number of progeny produced by each potential parent.

**Fig 1:**
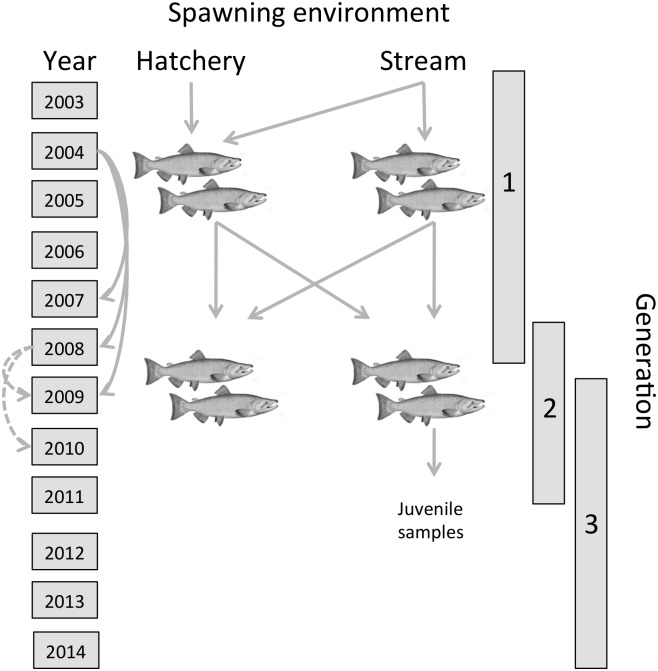
Illustration of the study design. Reproductive success (offspring per spawner) was measured in generation 2 by counting offspring in generation 3. Generation 3 was sampled only at the juvenile life-stages. Hatchery fish in generation 1 were produced in one of three ways: crossing two natural fish (H_NN_), a natural fish and a prior generation hatchery fish (H_HN_), or two prior generation hatchery fish (H_HH_). Fish in the population returned to spawn primarily at ages 3, 4 and 5.

Our study encompassed portions of three generations, but focused primarily on the second generation (Fig 1): generation 1 consisted of fish spawned in the hatchery, some of which were prior generation hatchery origin fish and some of which were newly captured natural origin fish; generation 2 consisted of the naturally spawning adult progeny of the generation 1 hatchery fish and of natural origin fish returning to spawn in the same years; generation 3 consisted of the natural origin juvenile progeny of generation 2. Broodstock origin (H_HH_, H_HN_, H_NN_) of hatchery fish in generation 2 was determined from differential tagging prior to release and, in the case of lost tags, from parentage assignment to generation 1. The reproductive success of individuals in generation 2 was estimated by counting the number of progeny from generation 3 genetically assigned to each fish in generation 2 using parentage analysis.

### Study population

**Fig 2:**
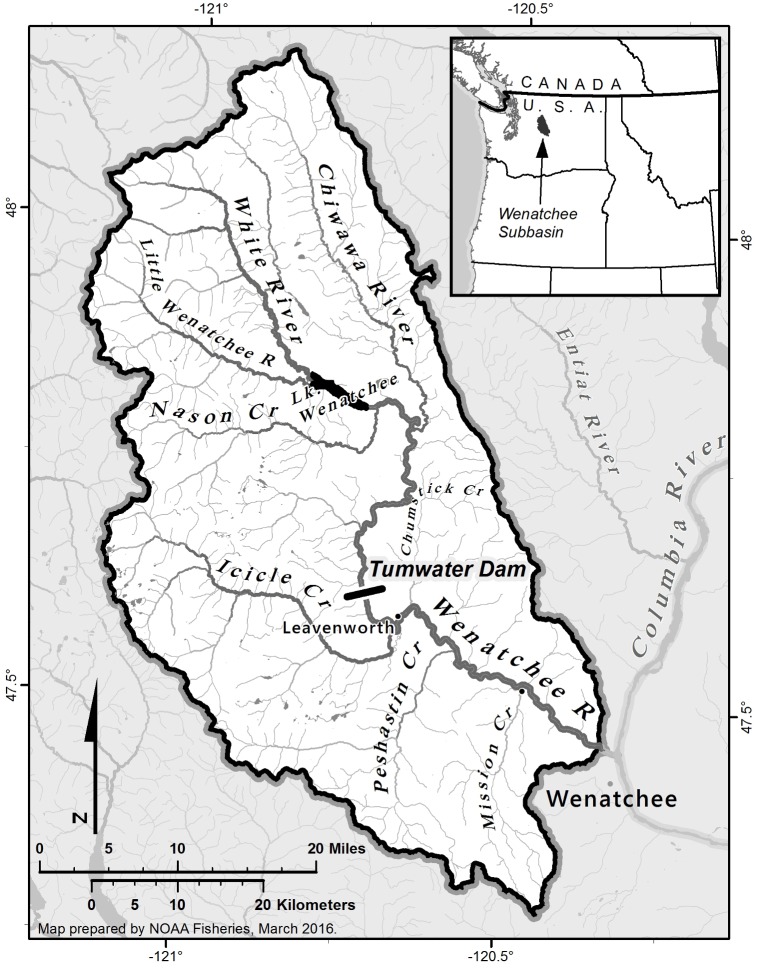
Map of the study area. Adults were sampled at Tumwater Dam. Juveniles were sampled in the Chiwawa River, Nason Creek, and Wenatchee River.

Our study population was the group of steelhead that spawn in the upper Wenatchee River watershed, upstream of Tumwater Canyon (Fig 2). The population has a complex life-history pattern typical of Interior Columbia Basin steelhead [24]. Spawning occurs in late spring in the Wenatchee River and several of its major tributaries, primarily the Chiwawa River and Nason Creek (Fig 2). Juveniles typically rear in the Wenatchee watershed for 1–3 years (most commonly 2 years) prior to undergoing a spring migration downstream to the Columbia River and thence to the Pacific Ocean. The fish typically spend 1–2 and occasionally up to 4 years in the ocean, before returning again to their natal stream to spawn [25]. Most of the spawning population returns during the summer and fall 5–10 months prior to spawning, overwintering in the Upper Wenatchee watershed. A portion also overwinters in the Columbia River or lower Wenatchee River and returns to the upper Wenatchee in the spring immediately prior to spawning.

The Wenatchee River steelhead population is one of four extant populations comprising the Upper Columbia River steelhead Distinct Population Segment (DPS), which is listed as “threatened” under the U.S. Endangered Species Act [1, 25]. Releases of hatchery steelhead into the Wenatchee River began in the mid-20^th^ Century and continue to the present time. The source populations of the released fish have varied over time, but have primarily consisted of fish originating from the Upper Columbia region, and since 1998 only from the Wenatchee River [25, 26].

### Sample collections

All work and associated sampling activities for both adults and juveniles were conducted in accordance with the Salmonid Disease Control Policy of the Fisheries Co-Managers of Washington State and the terms and conditions of Endangered Species Act Section 10(a)(1)(A) Permit 1395 to the Washington Department of Fish and Wildlife issued by the National Marine Fisheries Service. WDFW and NMFS do not require or provide for review by an Institutional Animal Care and Use Committee for research projects focused solely on fish, but the effects associated with sampling procedures and related activities were analyzed in the effects analysis of the Biological Opinion for Section 10(a)(1)(A) Permit 1395. All sampling was designed to be non-lethal, although the permit allowed for up 3% annual unintended mortality for juvenile samples. All captured fish were held in a recovery tank prior to release until they were fully recovered from anesthesia and swimming normally. Post-release survival of fish sampled as adults was evaluated during subsequent spawning ground surveys (see below); post-release survival of fish sampled as juveniles was evaluated through tag detections at downstream locations. In neither case, however, could the effects of tagging be distinguished from natural mortality.

#### Generations 1 and 2

Adult steelhead were collected annually from 2007 to 2011 in a trap situated off the fish ladder at Tumwater Dam (Fig 2) beginning in late June and ending the following May. The trap was operated 24 hours a day, 7 days a week, with rare interruptions during high flow events. During non-trapping periods including the winter months (December–February) the trap was not operated and passage was open, but video monitoring at a counting window in the ladder provided a count of the number of steelhead not trapped. During trapping periods, fish were diverted from the ladder into a collection chamber where water levels could be lowered to crowd fish into a hopper. The hopper was hoisted to a work platform and a low concentration of tricaine methanesulfonate (MS-222; 14 mg/L) was added before any fish were handled. Prior to sampling, steelhead were individually transferred from the hopper into a sampling tank (0.38 m^3^) containing a higher concentration of MS-222 (88 mg/L). Each steelhead trapped was measured (fork length) to the nearest cm, had scales collected for aging, was scanned with a portable ultrasound device to determine sex, had a small piece (~0.5 cm^2^) of caudal fin removed for genetic analysis, and was scanned for internal passive integrated transponder (PIT) tags. Each fish was also classified as either of hatchery or natural origin, based on the presence or absence of a hatchery mark (adipose fin clip, visible elastomer tag, or highly eroded fins). Here, we define a natural (or wild) fish as a fish whose parents spawned in nature, and a hatchery fish as fish whose parents spawned in a hatchery, in both cases regardless of prior generation ancestry. After sampling, fish were placed into a recovery tank and allowed to fully recover prior to being released upstream.

Hatchery fish were further categorized as H_HH_, H_HN_, or H_NN_ based on differential tagging prior to release and in a small number of cases by genetic parentage analysis of the generation 1 hatchery broodstock. The generation 1 hatchery broodstock were collected annually between July 1 and November 15 at Tumwater and Dryden Dams in the Wenatchee River. For our study, most generation 1 hatchery fish were spawned in 2004–2008, and the number of fish spawned ranged from 131 to 191 individuals per year [26]. Spawning typically consisted of single pair matings, with milt from one or two secondary males added after initial fertilization with the primary male. Spawning and subsequent supplementation protocols varied over time and are described in detail in [26]. Natural x natural crosses (resulting in H_NN_ fish in generation 2) were created in all years. Hatchery x hatchery crosses (resulting in H_HH_ fish) were only produced for the supplementation program in 2004 and 2005, and hatchery x wild (both reciprocal crosses) were produced in 2004–2007 [26]. Juvenile steelhead were initially reared separately based on cross type until they were externally marked in the adipose tissue posterior of the eye with a visible elastomer tag color coded to distinguish three cross types. Incubation and rearing occurred in a variety of Upper Columbia River hatchery facilities. The H_NN_ fish were initially reared at the Chelan Hatchery Complex (located on the Columbia River above Rocky Reach Dam near Chelan Falls, WA), while H_HH_ and H_HN_ were reared at the Eastbank Hatchery Complex (located near Rocky Reach Dam, 7 miles north of Wenatchee, WA). All cross types were reared during winter at the Turtle Rock Island Acclimation Facility (located downstream of Rocky Reach Dam) and released as smolts into the Wenatchee River watershed in May of each year as yearlings after an additional period of acclimation within the Wenatchee watershed. There were some differences among the cross types in acclimation location and time: H_NN_ fish were released primarily in Nason Creek, after an average of 145 days of acclimation, H_HH_ fish were acclimated for an average of 116 days prior to release into the Wenatchee River, and H_HN_ fish were acclimated for an average of 110 days before release into either the Chiwawa or Wenatchee Rivers [26].

All sampled steelhead were implanted with a passive integrated transponder (PIT) tag unless they already had a tag present. Recaptures of PIT tagged adults at PIT tag interrogation sites downstream of Tumwater Dam were removed from the pool of potential parents. A comprehensive system of instream PIT tag interrogation systems was installed at or near the mouth of all steelhead spawning tributaries during the study period [27]. The high proportion of steelhead that were PIT tagged (99%) provided a method to examine the distribution of hatchery and natural steelhead among spawning tributaries within the Wenatchee watershed. Each interrogation system was comprised of two arrays that spanned the channel. Detection probability was calculated based on the proportion of fish detected on the upper array that were also detected on the lower array. Fish were assigned to a spawning tributary based on the sequence of upstream detections during the spawning season.

#### Generation 3

Juvenile sampling occurred in conjunction with existing monitoring activities already ongoing in the Wenatchee Basin [26] using a variety of capture methods. Sampling was conducted between March and November of each year using a combination of angling and snorkel/seining and rotary smolt traps. Washington Department of Fish and Wildlife, Chelan County Public Utility District, and Yakama Nation personnel captured and released juvenile steelhead in each of the three major spawning areas upstream of Tumwater Dam (Nason Creek, Chiwawa River and Wenatchee River) and in a smolt trap in the lower Wenatchee River between the town of Leavenworth and confluence with the Wenatchee River (Fig 2). Regardless of capture method, captured fish were anesthetized in a solution of MS-222 (40–60 mg/L) prior to sampling. In order to determine brood year, each juvenile steelhead was scale sampled and age was determined from the scale annuli by the WDFW Scale Lab in Olympia, WA. Prior to release, a small portion of the distal dorsal lobe of the caudal fin (~0.1 cm^2^) was clipped for DNA analysis. All fish collected were also PIT tagged in the body cavity to prevent double sampling. Following sampling, fish were allowed to fully recover in 9 L buckets containing river water and then released in a calm part of the river near the sampling location. For fish sampled upstream of Tumwater Dam, a spatially representative subsample was randomly selected for parentage analysis based on the average number of redds surveyed in each tributary in each year (Hillman *et al*. 2015).

### Genotyping, parentage analysis and fitness estimation

Genomic DNA was extracted from samples using a Qiagen DNeasy kit and associated protocol. Parentage analysis between generations 2 and 3 was based on collecting genotypes at 96 single nucleotide polymorphism (SNP) loci (S1 Table) using a Fluidigm EP-1 genotyping system and data collection software version 4.1.1. Fluidigm Genotyping Analysis Software version 4.1.2 was used to process the resulting chip images. Limited genetic data compiled from other monitoring efforts [28] was available from a portion of generation 1, and was used to complement the tagging information to identify the hatchery broodstock types of generation 2 fish in a small number of cases where a fish lost a tag.

Pedigree estimation was conducted using likelihood methods [29] to evaluate parent-offspring relationships as implemented in the program FRANZ [30]. Only individuals with >48 genotyped loci were included in the analysis, and we assumed a per locus error rate of 1%. The predicted effectiveness of parentage assignments was evaluated using the program’s simulation function. To further evaluate the expected accuracy of the parentage assignments, we also conducted an analysis in which the adult returns in 2009, 2010, and 2011 were used as “progeny” for the parents spawning in the prior year in order to empirically determine the rate of false assignment when the true parents were known not to be in the sample. Because there was some potential uncertainty in the sex of the fish sampled at Tumwater Dam, the parentage analysis was conducted without considering the assigned sex of the spawners. In order to resolve any inferred sexing errors, assigned sexes of parentage pairs were compared and if a member of an apparent same sex pairing also appeared in a male/female pairing elsewhere in the data set, that member was assumed to have been sexed correctly and the other member of the same sex pairing had its sex designation changed.

Reproductive success was estimated as the number of juvenile progeny assigned to an individual, and was calculated separately for male and female parents. Lifetime fitness could not be estimated because adult progeny samples were not available. Relative reproductive success of hatchery fish compared to natural fish was calculated as the average reproductive success of the hatchery fish divided by that of natural fish, within years and sexes. Confidence intervals on relative reproductive success were estimated using a maximum likelihood method [31]. The statistical significance of the differences in mean number of progeny between hatchery and natural origin fish was tested using *t* tests. In addition, the effects of age, origin, size (fork length), migration date and spawning location on fitness were estimated using a general linear model with a negative binomial distribution and a log link function (glm.nb function in the MASS package of the R computer package version 3.2.1 [32]). Due to limited sample sizes and the very large number of potential interactions, we included only main effects in the model. Relationships between reproductive success, and the continuous variables of size and run timing were also evaluated and visualized using local polynomial regression as implemented in the LOESS function in R using the default parameters (span = 0.75, polynomial degree = 2).

## Results

A total of 6200 adult steelhead were captured, sampled and released above Tumwater Dam, excluding 715 fish that were collected for broodstock, killed as surplus, or returned below the dam prior to spawning (Table 1). For three of the four spawing years, a majority of the potential spawners were hatchery origin; in the fourth year >70% were natural origin. The annual proportion of migrating fish trapped varied slightly (98.7% - 99.9%) and over the entire study period 99.4% of the anadromous steelhead passing the dam were sampled. A small proportion (9%) of the returning hatchery fish could not be assigned to a broodstock cross type due to a combination of tag loss and lack of genotype information from their generation 1 parents (Table 1).

**Table 1:** Adult generation 2 steelhead by cross type that were captured, sampled and released to spawn above Tumwater Dam. Nat. refers to natural origin fish, and H_U_ refers to hatchery fish whose cross type was not known.

	Males					Females				
Year	Nat.	H_HH_	H_HN_	H_NN_	H_U_	Nat.	H_HH_	H_HN_	H_NN_	H_U_
2008	258	65	188	260	34	225	21	106	125	10
2009	169	33	280	147	96	182	59	293	185	108
2010	401	1	529	323	30	379	1	331	212	29
2011	318	0	1	172	0	489	0	0	139	1
Total	1146	99	998	902	160	1275	81	730	661	148

The most common life-history pattern for natural origin fish was to spend 2 years in freshwater prior to ocean migration followed by 1–2 years of ocean residence (S2 Table) before returning to spawn. Accounting for an additional winter in freshwater as an adult prior to spawning, most natural steelhead in this population spawned at ages 4 or 5. A small number (42; 0.7%) were repeat spawners that were making their second spawning migration after having returned to the ocean after their first spawning migration. In contrast to natural fish, nearly all hatchery fish spent only 1 year in freshwater (reflecting the hatchery program’s yearling release strategy–[26]), followed by 1–2 years in the ocean, resulting in primarily 3–4 year old spawners (S2 Table).

A majority of fish passed Tumwater Dam during the summer season and overwintered in the Wenatchee Basin, but a significant minority passed Tumwater Dam in the spring (S1 Fig). The proportion of summer versus spring returns was similar for natural fish and H_HH_, and H_HN_ hatchery fish, but the H_NN_ hatchery fish had a notably higher proportion of spring returns than the other groups (Table 2). Based on PIT tag detections at Columbia River Dams downstream of the Wenatchee River, the entire population, including fish passing Tumwater Dam in the spring, enters freshwater during the summer (ARM, unpublished data). The bi-modal distribution of run time at Tumwater Dam therefore reflects local differences in migration and overwintering location rather than return time from the ocean.

**Table 2:** Proportions of spring and summer run timing for natural origin steelhead (Nat.) and the three hatchery broodstock cross types. The proportion returning in each season differs significantly among categories for both males and females (chi-square contingency tests, *p* < 0.0001).

	Males				Females			
	Nat.	H_HH_	H_HN_	H_NN_	Nat.	H_HH_	H_HN_	H_NN_
Summer	0.741	0.687	0.811	0.541	0.831	0.877	0.858	0.649
Spring	0.259	0.313	0.189	0.459	0.169	0.123	0.142	0.351
n	1146	99	998	902	1275	81	730	661

For the dominant ocean age classes, there were significant differences in length between the spawner categories (natural fish and the three hatchery fish cross types) for both males and females, but the differences were relatively small, particularly for fish that only spent 1 year in the ocean (S2 Fig). For all spawner categories of both sexes, fish that spent 2 years in the ocean were significantly larger than 1 ocean fish. There were notable differences in spawning location among natural fish and the three categories of hatchery fish (Table 3). Except for H_NN_ fish, in all four years the most common assigned spawning location was “Other”; i.e., fish that were not detected in either of the two actively monitored streams (Nason Creek and the Chiwawa River). The fish in this “Other” category consisted of fish that presumably spawned in the Wenatchee River and small spawning streams such as Chiwaukum Creek and the White River [27] (Fig 2), as well any fish that died prior to detection on a PIT tag array or were otherwise not detected. Of the actively monitored streams, natural fish were detected in Nason Creek somewhat more frequently than in the Chiwawa River, with a ratio of ~ 3:2 between the two streams. H_HH_ hatchery fish were rarely detected in either monitored tributary, consistent with their release location in the Wenatchee River. H_HN_ were detected more frequently in the Chiwawa River than in Nason Creek, while for H_NN_ the reverse was true (Table 3). The observed distribution of hatchery steelhead was consistent with their juvenile release locations (see methods).

**Table 3:** Proportion and samples size of natural (Nat.) and the three types of hatchery steelhead and unknown hatchery fish detected as spawning in the Chiwawa River, Nason Creek, or Other/unknown spawning location.

	2008				2009				2010				2011			
stream	Nat.	H_HH_	H_HN_	H_NN_	Nat.	H_HH_	H_HN_	H_NN_	Nat.	H_HH_	H_HN_	H_NN_	Nat.	H_HH_	H_HN_	H_NN_
Chiwawa	0.091	0.012	0.190	0.034	0.205	0.033	0.237	0.072	0.218	1.000	0.357	0.073	0.197	-	0.000	0.074
Nason	0.155	0.047	0.065	0.377	0.285	0.011	0.065	0.633	0.264	0.000	0.051	0.654	0.322	-	0.000	0.521
Other	0.754	0.942	0.745	0.590	0.510	0.957	0.698	0.295	0.518	0.000	0.592	0.273	0.481	-	1.000	0.405
n	483	86	294	385	351	92	573	332	780	2	860	535	807	0	1	311

Of the 6200 potential spawners, 6165 were successfully genotyped at >48 loci, and 95% were genotyped at >90 loci. We also genotyped a total of 5497 juveniles >48 loci (94% > 90 loci); n = 1201, 1209, 2119, and 968 for the 2008–2011 broodyears, respectively. Most juveniles were sampled in the mainstem Wenatchee River (48%), followed by Nason Creek (33%) and the Chiwawa River (19%), with small contributions from other tributaries. Average observed and expected heterozygosities for the nearly 12,000 generation 2 and generation 3 individuals genotyped were 0.408 and 0.417, respectively, with an overall *F*_IS_ value of 0.023. The lower than expected proportion of heterozygotes is likely caused by minor population substructure, both within the watershed and over time. Across the four years, *F*_ST_ ranged from 0.0030 between natural fish and H_NN_ fish, to 0.0192 between H_HH_ and H_NN_ fish, with comparisons involving H_HH_ fish greater than other comparisons (Table 4). Theoretical exclusion probabilities calculated using FRANZ for the 96 loci combined were: 0.999884 for the first parent, 1.0000000 for the second parent, and 1.0000000 for a parent pair. Simulation results from the FRANZ program indicated that >99% of true parent/offspring pairs were expected to mismatch at <3 loci, while >99% of unrelated pairs would mismatch at >3 loci. Similarly, the simulations indicated that >99% of true parent-pair/offspring would mismatch at <3 loci, whereas >99% of offspring/mother/unrelated triplets would mismatch at least 7 loci.

**Table 4:** Estimates of *F*_ST_ among the four hatchery types of generation 2 hatchery fish and natural fish.

Comparison	*F*_ST_
H_HH_ / H_HN_	0.0104
H_HH_ / H_NN_	0.0192
H_HH_ / Nat.	0.0132
H_HN_ / H_NN_	0.0074
H_HN_ / Nat.	0.0037
H_NN_ / Nat.	0.0030

The vast majority of the 2115 inferred matings were between fish with opposite sex designations. In the initial analysis, there were 169 progeny assigned to apparent female-female pairings, and 231 to apparent male-male pairings. To resolve these discrepancies, 107 originally designated females had their sex designation changed to male, and 104 originally designated males were changed to females. In this way, all parent-pair assignments were between a male and a female. In total, 66% of offspring assigned to 2 parents, 15% to a dam only, 6% to a sire only, and 13% to neither parent. The median posterior probability for the parentage assignments was 0.998, and 84% were > 95%. The portion of assignments with < 3 mismatching alleles were 95.5% and 99% for the two and single parent assignment, respectively.

In the three negative control analyses in which adults from one year were treated as “progeny” from parents in the prior year, the apparent rates of false assignment to single parents were 43%, 23%, and 35%, for the 2009, 2010 and 2011 adult returns years, respectively. The apparent rates of false assignment to parent pairs were 4.4%, 0.6%, and 3.1%, respectively. Because of the potentially high rate of false assignment to single parents, we conducted all subsequent analyses twice: once using all progeny assignments, and once using only the subset of assignments to parent pairs. The parameter estimates and *p-*values obtained from the two analyses were very similar, so only the results from the parent-pair assignments (which are likely to be more reliable) are reported below.

**Fig 3:**
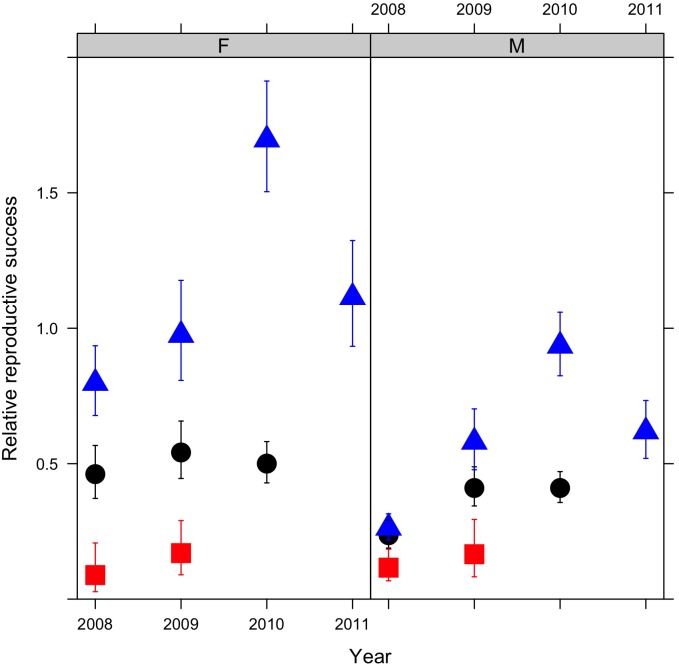
Relative reproductive success (RRS) and 95% confidence intervals of hatchery females (F) and males (M) compared to natural fish. Hatchery fish are further categorized by their broodstock origin the prior generation (H_HH_ = hatchery x hatchery (red squares), H_HN_ = hatchery x natural (black circles), H_NN_ = natural x natural (blue triangles)). In each case, RRS is calculated as the hatchery mean divided by the natural mean for fish spawning in the same year (Table 5).

Based on the progeny assignments, there were large differences in relative reproductive success (RRS) between natural and hatchery fish (Fig 3, Table 5). H_HH_ hatchery had particularly low RRS, with only 15–20% of the per capita progeny production of natural origin fish. H_HN_ were intermediate, with RRS values of ~50% for female and 25–43% for males. In contrast, female H_NN_ spawners had roughly equivalent, and in one year significantly higher, reproductive success compared to natural females, while H_NN_ male spawners had lower reproductive success than natural males in 3 of the four years.

**Table 5:** Mean progeny numbers and relative reproductive success (RRS) for hatchery and natural fish. The hatchery fish are further categorized by their broodstock origin the prior generation (H_HH_ = hatchery x hatchery, H_HN_ = hatchery x natural, H_NN_ = natural x natural). In each case, RRS is calculated as the hatchery mean divided by the natural mean for fish spawning in the same year.

Natural							
sex	year	n	mean	sd	RRS	95% CI	*p*-value
M	2008	258	2.120	3.691	—	—	—
M	2009	169	1.828	3.218	—	—	—
M	2010	401	1.424	2.571	—	—	—
M	2011	318	1.626	3.358	—	—	—
F	2008	225	2.147	2.934	—	—	—
F	2009	182	1.198	1.693	—	—	—
F	2010	379	1.443	2.071	—	—	—
F	2011	489	1.072	1.886	—	—	—
H_HH_							
M	2008	65	0.246	1.173	0.116	0.068–0.184	0.000
M	2009	33	0.303	1.132	0.166	0.082–0.294	0.000
M	2010	1	0.000	NA	NA	—	NA
M	2011	0	0.000	NA	NA	—	NA
F	2008	21	0.190	0.512	0.089	0.028–0.207	0.000
F	2009	59	0.203	0.581	0.170	0.090–0.290	0.000
F	2010	1	0.000	NA	NA	—	NA
F	2011	0	0.000	NA	NA	—	NA
H_HN_							
M	2008	188	0.500	1.298	0.236	0.188–0.292	0.000
M	2009	280	0.750	1.609	0.410	0.344–0.488	0.000
M	2010	529	0.584	1.532	0.410	0.357–0.471	0.000
M	2011	1	0.000	NA	NA	NA	—
F	2008	106	0.991	2.054	0.461	0.372–0.567	0.000
F	2009	293	0.648	1.315	0.541	0.445–0.657	0.000
F	2010	331	0.722	1.296	0.500	0.429–0.582	0.000
F	2011	0	0.000	NA	NA	NA	—
H_NN_							
M	2008	260	0.558	1.252	0.263	0.218–0.315	0.000
M	2009	147	1.061	2.055	0.580	0.478–0.702	0.011
M	2010	323	1.331	2.757	0.935	0.825–1.059	0.643
M	2011	172	1.006	2.175	0.619	0.520–0.733	0.014
F	2008	125	1.712	2.429	0.798	0.678–0.935	0.138
F	2009	185	1.168	1.823	0.975	0.807–1.177	0.869
F	2010	212	2.448	3.163	1.696	1.504–1.913	0.000
F	2011	139	1.194	2.163	1.114	0.933–1.324	0.545

Number of years spent in the ocean, length, season of return, day of return within either the spring or summer season, hatchery origin, and spawning location were all significant predictors of reproductive success, although parameter estimates and significance varied among years (Table 6). Consistent with the direct RRS estimates, for males each of the three hatchery cross types had lower reproductive success than natural origin males in all four years, and most parameters were significant. For female spawners, the H_HH_ and H_HN_ types had significantly lower reproductive success compared to natural females for all years tested, while the H_NN_ type had higher reproductive success in three years (2008, 2010 and 2011) and lower reproductive success in one year (2009; Table 6).

**Table 6:** Parameter estimates (with standard error and *p*-value: *<0.05, **<0.01, ***<0.001) for a negative binomial (log link) general linear model of offspring number as a function of fish traits. Traits include ocean age (1 or 2 years, with reference to age 1), hatchery cross type (with reference to natural origin spawners), fork length (cm), run timing season at Tumwater Dam (summer or spring, with reference to summer), run timing day of year at Tumwater Dam nested within each season, and spawning location (Chiwawa, Nason, or Other/Upper Wenatchee, with reference to Chiwawa).

	2008	2009	2010	2011
Males				
Intercept	1.726 (3.067)	-7.135 (3.544*)	-3.483 (2.255)	-2.906 (4.735)
Ocean age 2	-1.187 (0.400**)	-1.039 (0.308***)	-0.402 (0.236.)	-0.414 (0.378)
H_HH_	-1.337 (0.369***)	-1.597 (0.485***)	--	--
H_HN_	-0.889 (0.220***)	-0.604 (0.179***)	-0.420 (0.143**)	--
H_NN_	-0.483 (0.207*)	-0.276 (0.209)	-0.500 (0.157**)	-0.440 (0.226)
Length	0.133 (0.022***)	0.122 (0.018***)	0.094 (0.015***)	0.075 (0.019***)
Season (summer)	-9.274 (2.779***)	0.210 (3.279)	-3.193 (1.995)	-1.430 (4.624)
Location (Nason)	0.072 (0.301)	-0.914 (0.214***)	0.653 (0.169***)	0.342 (0.241)
Location (Other)	-0.126 (0.257)	-1.454 (0.185***)	-0.168 (0.146)	-0.902 (0.256***)
Run timing day (spring)	-0.033 (0.009***)	-0.001 (0.011)	-0.010 (0.006)	-0.007 (0.014)
Run timing day (summer)	-0.005 (0.003)	0.000 (0.002)	0.004 (0.002.)	-0.004 (0.003)
Females				
Intercept	0.164 (3.467)	-1.491 (3.185)	-0.740 (2.015)	2.382 (4.003)
Ocean age 2	0.384 (0.307)	-0.379 (0.273)	-0.032 (0.196)	-0.209 (0.329)
H_HH_	-1.923 (0.580***)	-1.015 (0.357**)	--	--
H_HN_	-0.541 (0.201**)	-0.399 (0.164*)	-0.421 (0.126***)	--
H_NN_	0.224 (0.192)	-0.227 (0.171)	0.227 (0.131)	0.261 (0.188)
Length	0.031 (0.020)	0.058 (0.017***)	0.033 (0.015*)	0.044 (0.017**)
Season (summer)	-1.510 (3.179)	-1.794 (2.937)	-1.554 (1.804)	-5.016 (3.821)
Location (Nason)	0.185 (0.291)	-0.136 (0.184)	0.462 (0.163**)	0.674 (0.180***)
Location (Other)	-0.383 (0.251)	-1.284 (0.161***)	-0.279 (0.137*)	-0.606 (0.187**)
Run timing day (spring)	-0.005 (0.010)	-0.005 (0.009)	-0.003 (0.006)	-0.018 (0.012)
Run timing day (summer)	0.000 (0.002)	0.002 (0.002)	0.003 (0.002.)	-0.005 (0.002**)

**Fig 4:**
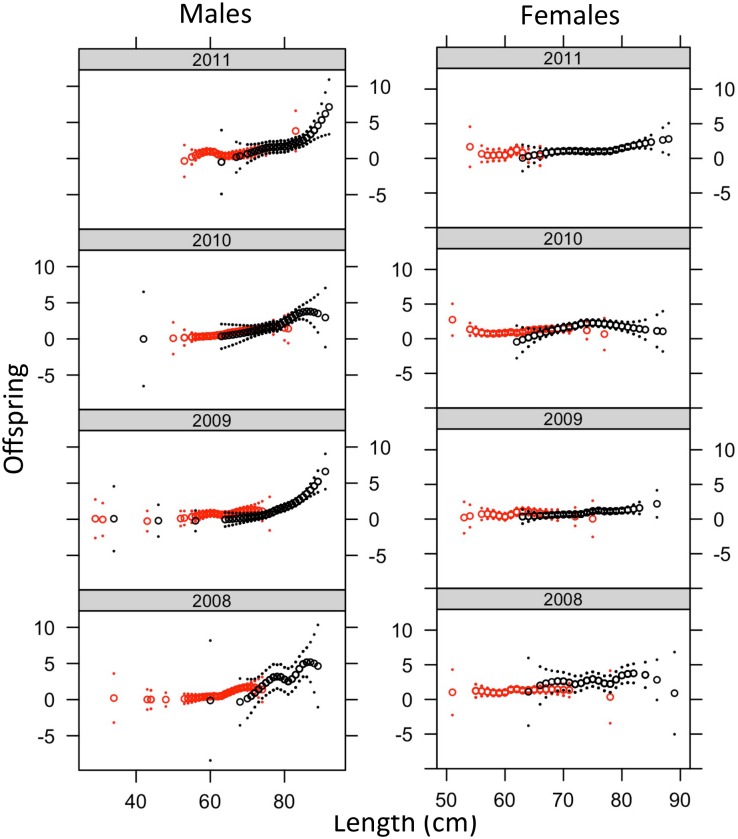
Smoothed relationships (with two standard error confidence bands) between reproductive success (offspring numbers) and length (cm), for ocean age 1 (red) and ocean age 2 (black) fish.

**Fig 5:**
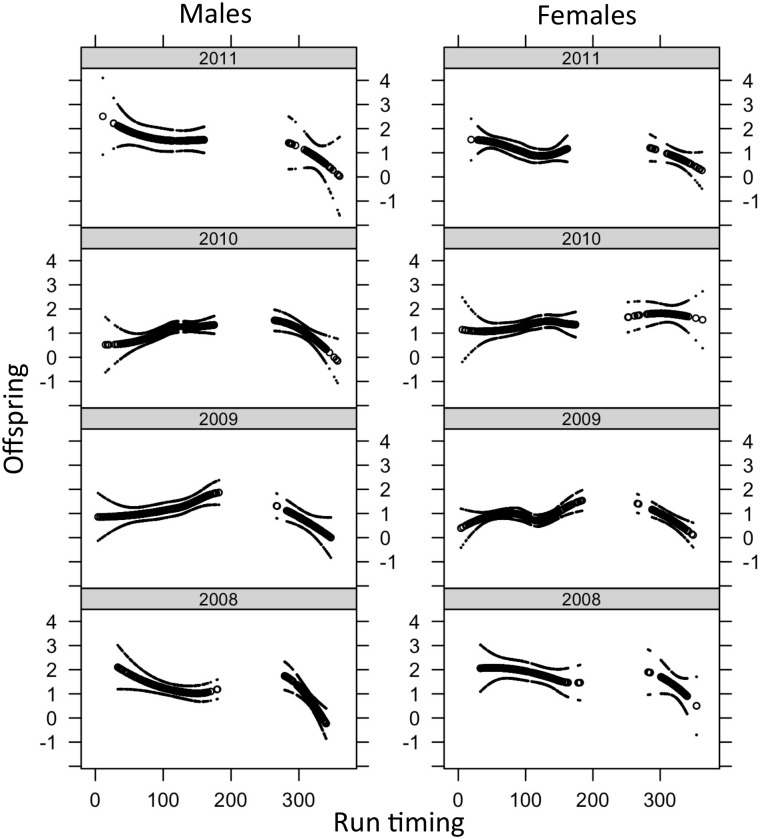
Smoothed relationships (with two standard error confidence bands) between reproductive success (offspring numbers) and run timing at Tumwater Dam (days after June 9).

For both sexes, larger size was consistently associated with higher reproductive success, but the effect was stronger for males than females (Table 6, Fig 4). For males, spending two years in the ocean had a negative effect on reproductive success at a given size in some years (Table 6, Fig 4). For males in 2008, early spring run timing was associated with higher reproductive success with a rapid drop-off in reproductive success during the spring season (Table 6), a pattern also visible in the local regression analysis in most years (Fig 5). For both sexes, in all four years spawning the Chiwawa River or Nason Creek was associated with higher reproductive success than in undetected locations (Table 6). For females in 2010 and 2011, spawning in Nason Creek was also associated with significantly higher reproductive success than spawning in the Chiwawa River. Males also had significantly higher success in Nason Creek compared to the Chiwawa River in 2010, but the opposite was true in 2009. Patterns of relative reproductive success among cross types were similar among locations, although smaller sample sizes resulted in wide confidence limits (S3 Fig).

## Discussion

Our finding that hatchery steelhead spawning in the Wenatchee River often had <50% of the reproductive success of natural origin steelhead is consistent with similar findings in other populations [12, 13]. The extremely low relative reproductive success of the H_HH_ hatchery fish is similar to what has been reported previously for long-term domesticated or non-local hatchery steelhead stocks [16, 33, 34]. This finding therefore reinforces previous management decisions to avoid the use of such stocks for use in supplementation programs [35–39].

Our study is the first study of steelhead to largely replicate the experimental design employed by an influential study of Hood River steelhead [17], comparing the reproductive success of H_HN_ and H_NN_ hatchery fish. Unlike similar recent studies of coho [23] and Chinook salmon [22] but like the Hood River study, we found that H_NN_ hatchery steelhead had substantially higher reproductive success than H_HN_ steelhead. The difference in reproductive success between the two types of hatchery fish was particularly pronounced for females. H_NN_ females had reproductive success similar to natural origin females while H_HN_ females’ reproductive success was only ~50% of that of natural origin females (Fig 3, Table 5).

The finding of differences between H_NN_ and H_HN_ steelhead in the Hood River population was interpreted as strong evidence for a heritable loss of reproductive success due to hatchery breeding and rearing. Specifically, the two types of fish were spawned, reared, and released in the same manner, differing in only whether they had one or two hatchery origin parents [17]. In the case of Wenatchee River steelhead, however, the three cross types experienced somewhat varying rearing, acclimation and release environments (see methods and [26]). We therefore cannot entirely rule out the possibility that differences in rearing conditions contributed to the differences in reproductive success observed among these groups.

Although fish originating from the three cross types experienced somewhat different environments, these environmental differences seem unlikely to explain all of the differences in fitness we observed. First, the pattern of reproductive success differences among the three cross types (H_HH_<H_HN_<H_NN_) is suggestive of a genetic effect associated with increasing natural ancestry and not with the documented environmental differences among the cross types. For example, H_HH_ and H_HN_ fish had similar acclimation durations, so that environmental difference seems unlikely to explain the difference in success between those two groups. Second, we were able to statistically evaluate the influence on reproductive success of differences in spawning location, a major environmental difference between the groups. Spawning location was in fact a significant factor influencing reproductive success in some years, but did not appear to explain the differences among the cross types or between hatchery and natural origin fish. In particular, origin was a significant effect in a model that also included spawning location (Table 6), and the patterns of relative reproductive success among the broodstock types were largely the same regardless of spawning location (S3 Fig). Thus, we conclude that genetic factors were important in determining differences in reproductive success among groups, although environmental factors may also have played a role.

The broodstock history of the hatchery origin fish used for the H_HH_ and the “H” portion of the H_HN_ crosses also suggests the possibility of genetic differences between these stocks and the natural fish that contributed to the H_NN_ cross. Starting in the mid-20^th^ century, there were extensive releases of hatchery steelhead into the Wenatchee River, and until 1998 none of the broodstock that produced these releases were collected from within the Wenatchee watershed but rather originated decades ago from collections of returning fish (initially of wild origin but subsequently primarily of hatchery origin) to Priest Rapids or Wells Dams on the Columbia River [24–26].

Starting in 1998, broodstock were collected only within the Wenatchee River system, but included ~50% returning hatchery fish. The Wenatchee River hatchery stock therefore originated as a mixture of natural fish from the Wenatchee River and hatchery fish with more mixed origins that included the Wenatchee but also other upper Columbia River steelhead populations such as those in the Entiat and Methow Rivers. Since 1998, the genetic influence of fish from this older Wells/Priest Dam hatchery stock has become diluted due to a priority for collecting primarily natural fish for broodstock [28], but it is possible that the H_HN_ and especially H_HH_ fish in our study still contained some genetic influence from the pre-1998 hatchery stock. If the natural population within the Wenatchee River maintained at least some genetic continuity with the wild population that existed prior to large-scale hatchery releases, genetic differences between the natural Wenatchee River fish and the hatchery fish used in the H_HH_ and H_HN_ crosses would not be surprising. Consistent with prior results [28], the patterns of genetic variation at the 96 loci used for parentage analysis indicated only minor allele frequency differences between hatchery and natural fish. However, the patterns of genetic differences are consistent with the patterns of relative reproductive success, with very little divergence between natural and H_NN_ fish and increasing (albeit still very small) levels of divergence between natural and H_HN_ and H_HH_ fish (Table 4).

Due to the uncertain history of the hatchery stock used to create the H_HH_ and H_HN_ broodstock, we also cannot necessarily conclude that the fitness differences among the three hatchery cross types and in particular the H_HN_ and H_NN_ types arose in only a single generation, as was suggested to have occurred in Hood River steelhead [17]. In particular, if the hatchery fish used to create the H_HH_ and H_HN_ crosses contained some fish originating from the older Wells stock, the differences in reproductive success between the H_NN_, H_HN_, and H_NN_ may be due to increasing the proportion of ‘natural’ genotypes into an older, diverged, hatchery stock rather than recent loss of fitness due to domestication selection on natural fish brought into the hatchery environment.

Hatchery steelhead have been released into the Wenatchee River for decades and are believed to have substantially altered the historical population structure and perhaps reduced the fitness of the natural population [24, 25, 40]. Our detection of substantial fitness differences between natural fish and fish with even a single hatchery parent suggests either that such differences can arise and be reversed in only several generations, or that the natural Wenatchee River steelhead population has retained a relatively high fitness despite a long history of hatchery releases in the watershed. The very low fitness of the H_HH_ fish suggests that it is plausible that the decades of relatively high returns of such hatchery stocks to the Wenatchee Basin had less impact than might be expected on the fitness of the natural population, because the hatchery fish may have had sufficiently poor fitness to greatly limit rates of gene flow into the natural population. This result is encouraging from a wild salmon recovery perspective as it suggests important genetic material may persist in natural populations despite decades of hatchery releases.

The finding that female H_NN_ hatchery fish had higher relative reproductive success than male H_NN_ hatchery fish continues a pattern seen in several other studies [12]. Similar to a recent study of Chinook salmon [8], we found no evidence of reduced reproductive success of H_NN_ females. The greater tendency of male hatchery fish to exhibit reduced reproductive success could be explained by a number of factors, such as greater sexual selection on males than females or a greater tendency for males to experience changes in epigenetic programming during hatchery rearing. Exploring the physiological causes for reduced reproductive success and the genetic or epigenetic architectures underlying these changes will require more detailed mechanistic study than we have done here.

We found a strong positive relationship between length and reproductive success, similar to what has been observed for Chinook salmon in the same river [22, 41] and steelhead elsewhere [42]. This relationship was stronger for males than for females (Table 6, Fig 4), but was significant for both sexes in most years. The relatively strong relationship between size and reproductive success for male steelhead is likely due to sexual selection, and is consistent with observations of spawning behavior in salmonids which show that larger males are more successful at obtaining mating opportunities than smaller males [43]. Interestingly, we found that in some years for the portion of the length distribution where they overlap, 1-ocean males tended to have slightly higher average reproductive success than males that spent two years in the ocean (Table 6, Fig 4). This observation suggests that smaller than normal length-at-age may be an indicator of poor fitness, particularly for males.

The relationship between reproductive success and run timing was more complex than the relationship with length, with a relatively flat relationship in the summer and a steeply declining relationship in the spring (Fig 5), although the latter was only significant in one year (Table 6). There are a number of previously identified factors that could contribute to poor fitness of late arriving steelhead, such as insufficient incubation time or insufficient resources for late emerging fry [42]. The lack of a consistent or strong relationship between return day within the summer time period and reproductive success is not surprising due to the long period between sampling and spawning for the summer-run fish.

The assignment rate of progeny to two parents (66%) in our study was notably higher than the 35–40% typical of other steelhead reproductive success studies, in which matings by unsampled resident trout have been implicated as a source of unassigned parents [16, 42, 44]. The higher rate of assignment to anadromous parents in our study compared to some other steelhead populations could therefore be caused by lower abundance of resident trout in the Wenatchee compared to other areas or to greater habitat segregation between life-history forms. Conspecific resident trout are present in the Wenatchee watershed [45], but are rarely observed in areas utilized by steelhead (ARM, unpublished data). A portion of the juveniles used in our study were sampled downstream of Tumwater Dam, however, and therefore could have been produced by unsampled anadromous parents spawning in lower Wenatchee River tributaries.

In summary, this study confirms that different degrees of prior-generation hatchery ancestry had a large impact on the subsequent reproductive success of naturally spawning steelhead. We found that incorporating natural fish into the broodstock is clearly beneficial for improving subsequent natural spawning success, even in a population that has a decades-long history of hatchery releases. Our results therefore generally support hatchery reform efforts aimed at integrating natural fish into hatchery broodstocks, particularly for hatchery programs where natural spawning by hatchery fish is expected to occur.

## Supporting Information

S1 Fig Distribution of run timing of steelhead potentially spawning above Tumwater Dam in days after June 9th. The majority of fish return in the summer prior to spawning in the spring.
(TIF)

S2 Fig Lengths (median, 1^st^ and 3^rd^ quantiles, range) of male and female steelhead potentially spawning above Tumwater Dam for the dominant ocean ages. For both males and females, ocean age and origin category were significant effects in a Gaussian general linear model (*p* < 0.05). Differences among years were not significant.
(TIF)

S3 Fig Relative reproductive success (RRS) and 95% confidence intervals of hatchery females (F) and males (M) compared to natural fish for three different spawning locations. Hatchery fish are further categorized by their broodstock origin the prior generation (H_HH_ = hatchery x hatchery (red squares), H_HN_ = hatchery x natural (black circles), H_NN_ = natural x natural (blue triangles)).
(TIF)

S1 Table SNP loci used for parentage analysis. (DOCX)

S2 Table Proportions and total numbers (n) of steelhead of different ages sampled at Tumwater Dam by cross type (Nat. = natural fish), based on scale analysis. (DOCX)
