# Supplementary figures and images for: Broodstock History Strongly Influences Natural Spawning Success in Hatchery Steelhead (Oncorhynchus mykiss)
Source: PLoS One. 2016 Oct 13;11(10):e0164801. doi: 10.1371/journal.pone.0164801 (PMC5063464; doi:10.1371/journal.pone.0164801)

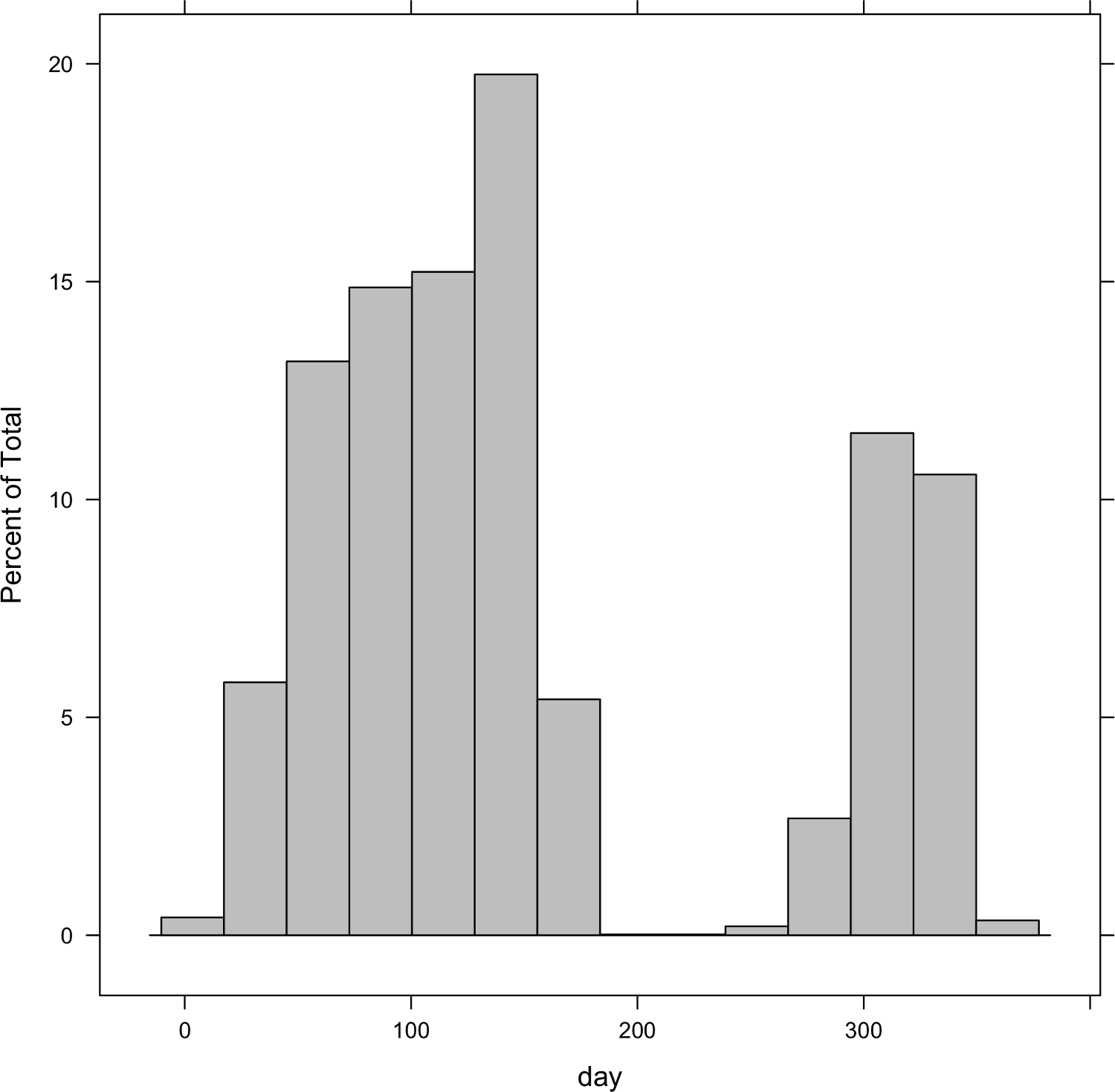

Supplement: S1 Fig — The majority of fish return in the summer prior to spawning in the spring. (TIF) [file pone.0164801.s001.tif]

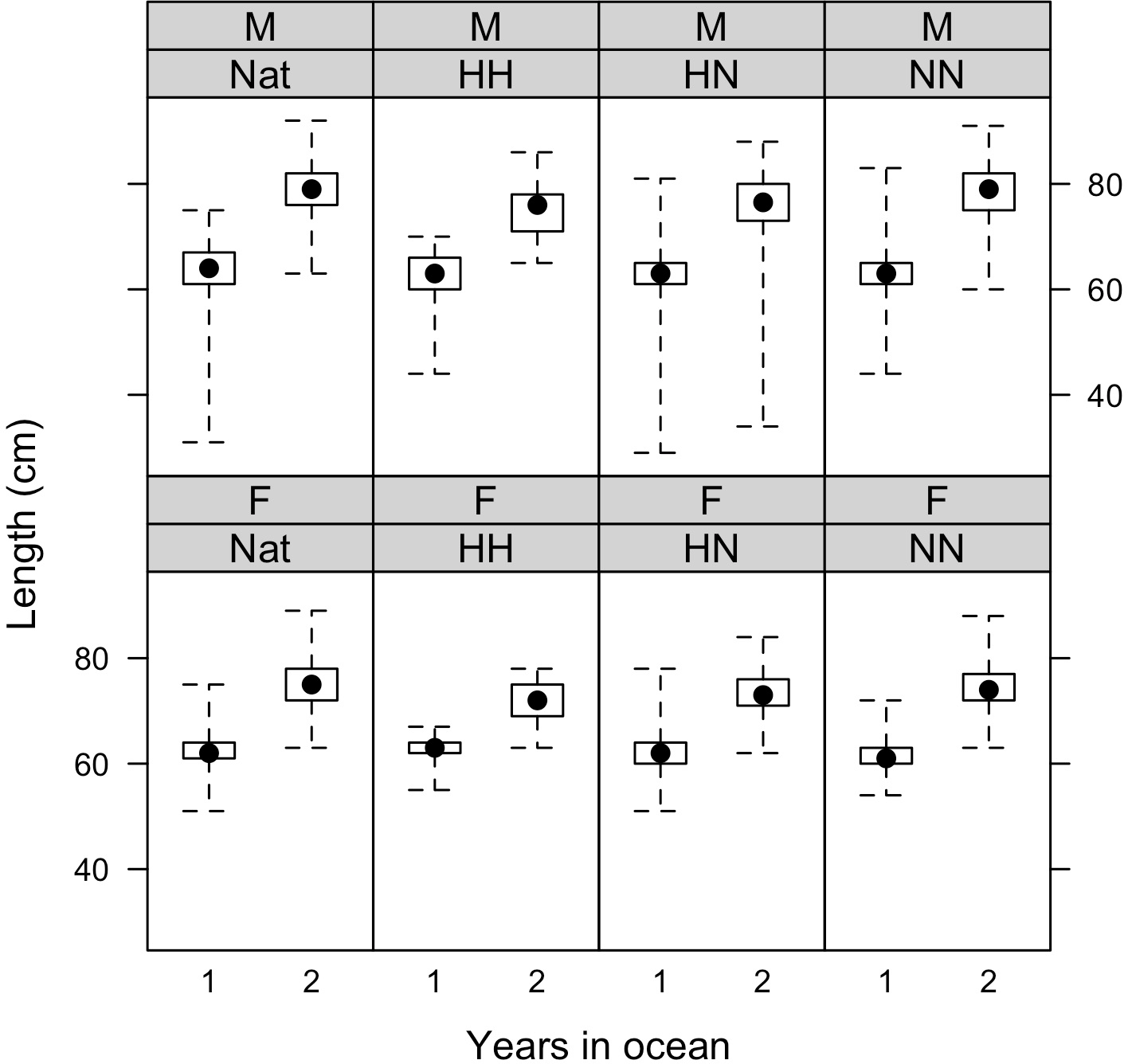

Supplement: S2 Fig — For both males and females, ocean age and origin category were significant effects in a Gaussian general linear model (p < 0.05). Differences among years were not significant. (TIF) [file pone.0164801.s002.tif]

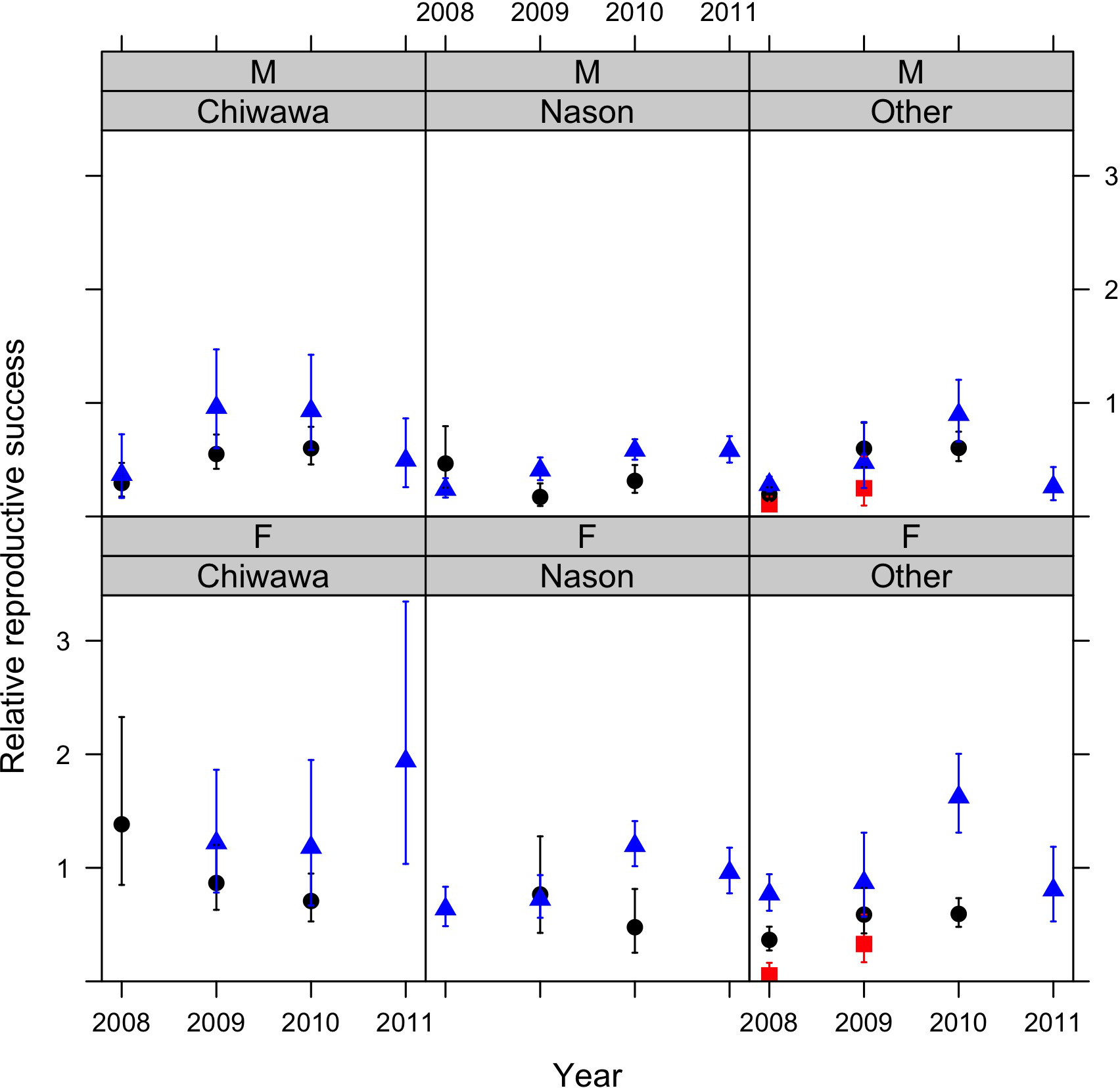

Supplement: S3 Fig — Hatchery fish are further categorized by their broodstock origin the prior generation (HHH = hatchery x hatchery (red squares), HHN = hatchery x natural (black circles), HNN = natural x natural (blue triangles)). (TIF) [file pone.0164801.s003.tif]
