# Supplementary material for: Broodstock History Strongly Influences Natural Spawning Success in Hatchery Steelhead (Oncorhynchus mykiss)
Source: PLoS One. 2016 Oct 13;11(10):e0164801. doi: 10.1371/journal.pone.0164801 (PMC5063464; doi:10.1371/journal.pone.0164801)
Supplement: S1 Table — (DOCX) [file pone.0164801.s004.docx]

S1 Table -- SNP loci used for parentage analysis

| Locus number | Locus name | Reference | Locus | Locus name | Reference |
| --- | --- | --- | --- | --- | --- |
| 1 | OMS00001 | [1] | 54 | Omy_116733_349 | [2] |
| 2 | OMS00006 | [1] | 55 | Omy_117242_419 | [2] |
| 3 | OMS00039 | [1] | 56 | Omy_117370_400 | [2] |
| 4 | OMS00048 | [1] | 57 | Omy_117815_81 | [2] |
| 5 | OMS00052 | [1] | 58 | Omy_118654_91 | [2] |
| 6 | OMS00053 | [1] | 59 | Omy_120950_569 | [2] |
| 7 | OMS00054 | [1] | 60 | Omy_121713_115 | [2] |
| 8 | OMS00056 | [1] | 61 | Omy_127236_583 | [2] |
| 9 | OMS00057 | [1] | 62 | Omy_127510_920 | [2] |
| 10 | OMS00062 | [1] | 63 | Omy_129870_756 | [2] |
| 11 | OMS00064 | [1] | 64 | Omy_130295_98 | [2] |
| 12 | OMS00071 | [1] | 65 | Omy_130524_160 | [2] |
| 13 | OMS00072 | [1] | 66 | Omy_95318_147 | [2] |
| 14 | OMS00078 | [1] | 67 | Omy_97660_230 | [2] |
| 15 | OMS00089 | [1] | 68 | Omy_98188_405 | [2] |
| 16 | OMS00090 | [1] | 69 | Omy_98409_549 | [2] |
| 17 | OMS00101 | [1] | 70 | Omy_99300_202 | [2] |
| 18 | OMS00105 | [1] | 71 | Omy_aldB_165 | [2] |
| 19 | OMS00106 | [1] | 72 | Omy_aromat_280 | [3] |
| 20 | OMS00109 | [1] | 73 | Omy_arp_630 | [4] |
| 21 | OMS00118 | [1] | 74 | Omy_aspAT_123 | [4] |
| 22 | OMS00120 | [1] | 75 | Omy_cd59_206 | Unpublished (J. DeKoning) |
| 23 | OMS00121 | [1] | 76 | Omy_colla1_525 | Unpublished (J. DeKoning) |
| 24 | OMS00132 | [1] | 77 | Omy_cox1_221 | [4] |
| 25 | OMS00154 | [1] | 78 | Omy_DAB_431 | Unpublished (J. DeKoning) |
| 26 | OMS00156 | [1] | 79 | Omy_DABb | [5] |
| 27 | OMS00175 | [1] | 80 | Omy_g12_82 | [6] |
| 28 | OMS00180 | [1] | 81 | Omy_gluR_79 | Unpublished (M. Campbell) |
| 29 | Omy_09AAD_076 | [2] | 82 | Omy_hsc715_80 | [3] |
| 30 | Omy_101832_195 | [2] | 83 | Omy_hsp47_86 | [7] |
| 31 | Omy_101993_189 | [2] | 84 | Omy_IL17_185 | Unpublished (J. DeKoning) |
| 32 | Omy_102505_102 | [2] | 85 | Omy_IL6_320 | Unpublished (J. DeKoning) |
| 33 | Omy_104519_624 | [2] | 86 | Omy_LDHB_2_e5 | [6] |
| 34 | Omy_105105_448 | [2] | 87 | Omy_metA_161 | Unpublished (J. DeKoning) |
| 35 | Omy_105385_406 | [2] | 88 | Omy_NaKATPa3_50 | [4] |
| 36 | Omy_105714_265 | [2] | 89 | Omy_nkef_241 | [4] |
| 37 | Omy_107031_704 | [2] | 90 | Omy_nkef_308 | [4] |
| 38 | Omy_107336_170 | [2] | 91 | Omy_Ogo4_212 | [4] |
| 39 | Omy_107786_314 | [2] | 92 | Omy_Ots249_227 | [4] |
| 40 | Omy_107806_34 | [2] | 93 | Omy_rapd_167 | [3] |
| 41 | Omy_108007_193 | [2] | 94 | Omy_stat3_273 | Unpublished (J. DeKoning) |
| 42 | Omy_109243_222 | [2] | 95 | Omy_u09_53_469 | [8] |
| 43 | Omy_109525_403 | [2] | 96 | Omy_u09_56_073 | [8] |
| 44 | Omy_109894_185 | [2] |  |  |  |
| 45 | Omy_110064_419 | [2] |  |  |  |
| 46 | Omy_110689_148 | [2] |  |  |  |
| 47 | Omy_111084_526 | [2] |  |  |  |
| 48 | Omy_111383_51 | [2] |  |  |  |
| 49 | Omy_112208_328 | [2] |  |  |  |
| 50 | Omy_114315_438 | [2] |  |  |  |
| 51 | Omy_114448_87 | [2] |  |  |  |
| 52 | Omy_114587_480 | [2] |  |  |  |
| 53 | Omy_114976_223 | [2] |  |  |  |

1. Sanchez CC, Smith TPL, Wiedmann RT, Vallejo RL, Salem M, Yao J, et al. Single nucleotide polymorphism discovery in rainbow trout by deep sequencing of a reduced representation library. BMC Genomics. 2009;10. doi: 10.1186/1471-2164-10-559. PubMed PMID: WOS:000272791700001.

2. Abadia-Cardoso A, Clemento AJ, Garza JC. Discovery and characterization of single-nucleotide polymorphisms in steelhead/rainbow trout, Oncorhynchus mykiss. Mol Ecol Resour. 2011;11:31-49. doi: 10.1111/j.1755-0998.2010.02971.x. PubMed PMID: WOS:000287485100004.

3. Aguilar A, Garza JC. Isolation of 15 single nucleotide polymorphisms from coastal steelhead, Oncorhynchus mykiss (Salmonidae). Mol Ecol Resour. 2008;8(3):659-62. doi: 10.1111/j.1471-8286.2007.02038.x. PubMed PMID: WOS:000254810300040.

4. Campbell NR, Overturf K, Narum SR. Characterization of 22 novel single nucleotide polymorphism markers in steelhead and rainbow trout. Mol Ecol Resour. 2009;9(1):318-22. doi: 10.1111/j.1755-0998.2008.02376.x. PubMed PMID: WOS:000262678900079.

5. Hansen MHH, Young S, Jorgensen HBH, Pascal C, Henryon M, Seeb J. Assembling a dual purpose TaqMan-based panel of single-nucleotide polymorphism markers in rainbow trout and steelhead (Oncorhynchus mykiss) for association mapping and population genetics analysis. Mol Ecol Resour. 2011;11:67-70. doi: 10.1111/j.1755-0998.2010.02978.x. PubMed PMID: WOS:000287485100006.

6. Stephens MR, Clipperton NW, May B. Subspecies-informative SNP assays for evaluating introgression between native golden trout and introduced rainbow trout. Mol Ecol Resour. 2009;9(1):339-43. doi: 10.1111/j.1755-0998.2008.02407.x. PubMed PMID: WOS:000262678900085.

7. Molecular Ecology Resources Primer Development C, Almany GR, De Arruda MP, Arthofer W, Atallah ZK, Beissinger SR, et al. Permanent Genetic Resources added to Molecular Ecology Resources Database 1 May 2009–31 July 2009. Mol Ecol Resour. 2009;9(6):1460-6. doi: 10.1111/j.1755-0998.2009.02759.x.

8. Limborg MT, Blankenship SM, Young SF, Utter FM, Seeb LW, Hansen MHH, et al. Signatures of natural selection among lineages and habitats in Oncorhynchus mykiss. Ecology and Evolution. 2012;2(1):1-18. doi: 10.1002/ece3.59. PubMed PMID: WOS:000312442000001.
