# Supplementary material for: Broodstock History Strongly Influences Natural Spawning Success in Hatchery Steelhead (Oncorhynchus mykiss)
Source: PLoS One. 2016 Oct 13;11(10):e0164801. doi: 10.1371/journal.pone.0164801 (PMC5063464; doi:10.1371/journal.pone.0164801)
Supplement: S2 Table — (DOCX) [file pone.0164801.s005.docx]

S2 Table -- Proportions and total numbers (n) of steelhead of different ages sampled at Tumwater Dam by cross type (Nat. = natural fish), based on scale analysis. For each age, the numbers left and right of the decimal point are the number of winters in fresh water prior to an ocean migration and the number of winters in the ocean prior to stream migration, respectively. An “S” indicates an additional winter in the ocean after a freshwater spawning run, and an “R” indicates an unknown number of winters in freshwater.

|  | Males | | | | |  | Females | | | | |
| --- | --- | --- | --- | --- | --- | --- | --- | --- | --- | --- | --- |
|  | Nat. | H_HH_ | H_HN_ | H_NN_ | H_U_ |  | Nat. | H_HH_ | H_HN_ | H_NN_ | H_U_ |
| 1.0 | 0 | 0 | 4 | 0 | 0 |  | 0 | 0 | 0 | 0 | 0 |
| 1.1 | 44 | 61 | 705 | 539 | 40 |  | 11 | 21 | 323 | 211 | 10 |
| 1.1S | 0 | 0 | 0 | 0 | 0 |  | 0 | 0 | 1 | 0 | 0 |
| 1.1S1 | 0 | 0 | 1 | 1 | 1 |  | 0 | 0 | 2 | 2 | 2 |
| 1.2 | 57 | 31 | 239 | 287 | 84 |  | 71 | 53 | 371 | 382 | 108 |
| 1.3 | 0 | 0 | 2 | 0 | 0 |  | 1 | 0 | 0 | 0 | 0 |
| 1.S | 0 | 0 | 1 | 0 | 0 |  | 0 | 0 | 0 | 0 | 0 |
| 2 | 0 | 0 | 0 | 2 | 0 |  | 0 | 0 | 0 | 0 | 0 |
| 2.1 | 421 | 0 | 4 | 9 | 11 |  | 277 | 0 | 1 | 0 | 3 |
| 2.1S | 0 | 0 | 0 | 0 | 0 |  | 4 | 0 | 0 | 1 | 0 |
| 2.1S1 | 2 | 0 | 0 | 0 | 0 |  | 7 | 0 | 0 | 0 | 1 |
| 2.2 | 307 | 0 | 6 | 4 | 4 |  | 559 | 0 | 1 | 7 | 6 |
| 2.2S | 0 | 0 | 0 | 0 | 0 |  | 0 | 0 | 0 | 0 | 1 |
| 2.2S1 | 1 | 0 | 0 | 0 | 0 |  | 3 | 0 | 0 | 0 | 0 |
| 2.3 | 4 | 0 | 0 | 0 | 0 |  | 4 | 0 | 0 | 0 | 0 |
| 2.S | 0 | 0 | 2 | 0 | 0 |  | 0 | 0 | 0 | 0 | 0 |
| 3 | 2 | 0 | 0 | 0 | 0 |  | 0 | 0 | 0 | 0 | 0 |
| 3.1 | 132 | 0 | 0 | 0 | 3 |  | 100 | 0 | 0 | 0 | 0 |
| 3.1S | 0 | 0 | 0 | 0 | 0 |  | 1 | 0 | 0 | 0 | 0 |
| 3.1S1 | 0 | 0 | 0 | 0 | 0 |  | 0 | 0 | 0 | 0 | 1 |
| 3.2 | 49 | 0 | 0 | 1 | 1 |  | 115 | 0 | 0 | 2 | 2 |
| 3.2S1 | 0 | 0 | 0 | 0 | 0 |  | 1 | 0 | 0 | 0 | 0 |
| 3.3 | 2 | 0 | 0 | 0 | 0 |  | 0 | 0 | 0 | 0 | 0 |
| 4 | 2 | 0 | 0 | 1 | 0 |  | 0 | 0 | 0 | 0 | 0 |
| 4.1 | 1 | 0 | 0 | 0 | 0 |  | 2 | 0 | 0 | 0 | 0 |
| 4.2 | 1 | 0 | 0 | 0 | 0 |  | 1 | 0 | 0 | 0 | 0 |
| R | 1 | 0 | 0 | 3 | 0 |  | 1 | 0 | 0 | 0 | 0 |
| R.1 | 59 | 5 | 24 | 34 | 4 |  | 31 | 0 | 10 | 9 | 0 |
| R.1S1 | 0 | 0 | 0 | 0 | 0 |  | 2 | 0 | 1 | 1 | 0 |
| R.2 | 42 | 2 | 10 | 8 | 11 |  | 79 | 7 | 20 | 40 | 14 |
| R.2S | 0 | 0 | 0 | 0 | 0 |  | 1 | 0 | 0 | 1 | 0 |
| R.3 | 2 | 0 | 0 | 0 | 0 |  | 0 | 0 | 0 | 0 | 0 |
| <NA> | 17 | 0 | 0 | 13 | 1 |  | 4 | 0 | 0 | 5 | 0 |
